# The Hospital. Nursing Mirror

**Published:** 1898-07-02

**Authors:** 


"The Hospital" Nursing Mirror.

Being the Nursing Section of "The Hospital."

[Contributions for this Section of "The Hospital" should be addressed to the Editor, The Hospital, 28 & 29, Southampton Street, Strand, London, W.C., and should have the word "Nursing" plainly written in left-hand top corner of the envelope.]

### News from the Nursing World.

### DUBLIN'S COMMEMORATION OF THE DIAMOND JUBILEE.

The fund collected in Dublin, for training district nurses, in commemoration of the sixtieth year of Her Majesty's reign has proved an unqualified success. A large sum has been raised, which will be divided into three equal portions. One will be paid to St. Patrick's Nursing Home, another to St. Lawrence's Catholic Home for Nurses, whilst the committee will retain the control of the third for the training and maintenance of the Queen's Jubilee Nurses in other parts of Ireland. The poor require much teaching—the Irish poor even more than the English—and the effect of the elementary education now compulsory in the Board schools, backed up by a knowledge of personal hygiene inculcated by trained nurses should, in the course of a few years, completely change for the better the condition of the peasantry.

### THE PRESS BAZAAR.

We were glad that The Hospital Stall proved a haven of rest for many weary nurses who worked so energetically at "The London Hospital Stall." The Hon. Sydney Holland, chairman of the hospital, was most generous in his entertainment of the nurses, whom he sent up in parties to enjoy strawberries and cream, and, above all, a rest amongst the charming surroundings of the fruit stall. One nurse from King's, who had been selling programmes all day without any refreshment, entered the sanctum of the room devoted to The Hospital with real delight, and expressed herself delighted with the entertainment the stallholders were pleased to be able to afford her. Later on in the evening two beautiful boxes of fruit were sent to Charing Cross Hospital.

### DISTRIBUTION OF THE AWARDS OF THE NATIONAL HEALTH SOCIETY.

H.R.H. Princess Christian distributed on June 11th the diplomas, medals, and certificates gained by the candidates successful at the various examinations held during the year under the auspices of the National Health Society. The presentations were made at Grosvenor House, kindly lent by the Duke of Westminster, who took the chair, and remarked at the opening that for twenty-one years he had been President of the society. He spoke of the work being done by the National Health Society in teaching hygiene and sanitation to the masters of regiments stationed at Aldershot—teaching undertaken at the request of H.R.H. the Duke of Connaught. Sir J. Crichton Browne, Sir A. Arnold, and the Rev. C. J. Ridgway were among the speakers.

### LITTLESTONE ON-SEA CONVALESCENT HOME.

The gift of a freehold site having been promised to the committee of the Littlestone-on-Sea Convalescent Home for Women and Girls, a bazaar was held in St. Martin's Town Hall, Charing Cross, on Wednesday and Thursday, June 29th and 30th, in aid of funds for the purpose. Lady Russell Reynolds performed the opening ceremony on the first day, and Lady Owen Roberts on the second. Various entertainments were provided, and the stalls prettily arranged. This charity was established in 1890, when a villa with accommodation for twelve beds was rented. It has outgrown the space at its command, and it is hoped that it will be possible to provide a ward for mothers with infants in the new building. The annual report can be obtained from the Hon. Director, at the Office of the Home 9, Farringdon Avenue, E.C. A contribution of £1 1s. entitles the subscriber to send a patient to the home for a fortnight.

### VOLUNTEER NURSES.

All American nurses seem smitten with a desire to emulate Florence Nightingale. Offers of nursing service come from all quarters, and the Government has only to ask in order to have any number of trained nurses. The Nurses' Associated Alumnæ, which has a membership of 2,000 trained nurses, passed a unanimous resolution at its annual meeting held recently at New York placing the organisation at the service of the Medical Department of the Army, which resolution was duly forwarded by the president, Miss Hunter, of Baltimore. The Boston branch of the American Red Cross Nurses, composed entirely of graduate nurses, is now under military instruction, and ready to be sent to the front at a moment's notice. English nurses, therefore, who desire to quit the more peaceful if not less dangerous spheres of hospital and private work for the battle field may put all idea of being able to do as they wish out of their minds. There is no room for them. Should such need comfort, let them remember that if all be not gold that glitters, neither is fame always to be found on the warpath. According to all accounts nurses and war correspondents are finding the Spanish-American hostilities exceedingly dull, the only thing apparently to be done under the circumstances being to wait patiently for an indefinite period.

### A USEFUL LEAFLET.

By far the greatest obstacle to progress in any direction whatsoever is the physical and mental inertia of the average mortal. True there are beings who are for ever busy; but, as a rule, they are busy along the line of the least resistance. People who have to work know what a hindrance the unceasing activity of the thoughtless is; and how often does mental fidget "let off" all its energy in words, exhausting the store of others in the process. In fact most people, however energetic they may seem, like to have their thinking done for them, and the result clearly and neatly stated for their use without any more bother. The Irish Workhouse Reform Association has very sensibly taken this peculiarity of the majority into account, and has "thought out" the Local Government Board order and drawn it up into the form of definite rules for the guidance of the nurses in the employ of the poor law. These programmes of duty are distributed to the nurses, who by their aid not only know what they must do themselves, but also what services they may claim from their pauper assistants. We can suggest an improvement, namely, a leaflet for the instruction of guardians, with the extent and limits of their powers under the new order equally neatly summed up. It is quite possible that the saving in money alone from such a plan would pay the difference between the salaries of the trained nurses and their untrained predecessors.

### WORKING LADIES' GUILD.

The annual meeting of the Working Ladies' Guild, 251, Brompton Road, was held on Tuesday at 66, Pont Street, S.W., by the kind permission of Lord and Lady Lawrence. Canon Pennefather, Vicar of Kensington, presided. Mrs. Creighton, Mrs. Locker Lampson, and others addressed the meeting. The treasurer's statement showed that during the past seven years the Guild, irrespective of its branches, had paid to workers, and given away in pensions, relief in sickness, and educational grants, £26,000. The Guild helps widows and unmarried ladies alone, who must be recommended by an associate to whom they are personally known; 137 engagements of all sorts were made through its registry during the past year, although increasing difficulty is felt in finding engagements, especially for the middle-aged. The Guild has this year attained its majority, and numbers 1,700 associates and four branches, besides eight groups of associates who meet in various parts of London to consider and help the cases brought before them for relief. Mrs. Creighton spoke of the paramount importance of training ladies for employment, and instanced the openings which existed for lady nurses for children, and for elementary teachers in national schools.

### A STEP IN ADVANCE.

The trustees of the Peter Brough District Nursing Home, Paisley, in order to encourage their nurses to join the Royal National Pension for Nurses, and so make provision for age and sickness, now pay half the premiums for the nurses on their staff.

### THE AMERICAN AMENDMENT.

The clause authorising the Red Cross Association of all countries to intervene in cases of disaster with which the local authorities of the district might be unable to cope, which was unanimously inserted in the articles of the Red Cross Convention, is known as the "American Amendment." By virtue of it, Miss Barton has been actively engaged in various works of mercy during the last twenty years, the consequence of which is that many Americans have lost sight of the primary object of the Red Cross Association. Her recent expedition to Cuba was at first welcomed by the Spanish, but upon the political relations becoming strained the leaders were advised to leave the island. Although the provisions and supplies were far from being exhausted, the remainder was withdrawn, because in all probability it would have been used for the Spanish soldiers, and not for the starving Cubans for whom it was intended. Miss Barton is reported to have said that the Spanish military authorities regard the association as interlopers, seeking to interfere in matters that do not concern them. They also believe that their own military medical staff is sufficient for the campaign.

### THE CATHEDRAL NURSE AND LOAN SOCIETY.

The annual meeting of the Cathedral Nurse and Loan Society took place in the Union Offices, Pilgrim Street, Newcastle, when the Vicar of Newcastle, Canon Gough, read the fifteenth report. About six new cases are taken up by the society, and fifty visits made by the nurses daily, whose work is supplemented by a sick kitchen. Convalescent homes are maintained in Hexham for girls and women, whilst men are sent into lodgings. Numerous branches in outlying districts are in satisfactory working order. The blot on the report lies in the balance sheet, which showed a deficit of £121 in an expenditure of £1,709. Much good work has been done; more remains to be done; and with influential and sympathetic friends the society ought not to be hindered for want of money.

### A WORD OF WARNING.

Familiarity breeds contempt; nurses being no more exempt from this rule than others. The constant handling of ominous blue bottles labled "poison" is apt to wear off the wholesome awe with which those unaccustomed to the sick room regard them. The sad death of Nurse Gladys Wilson Hewett at Pensnett Vicarage from an overdose of chloroform, a drug which she had got into the habit of inhaling, is a painful example of this. Nurses in the full vigour of strength may perhaps regard the poor girl with a sort of contemptuous pity, and think that, though, of course, there is a danger to the foolish and careless, it passes them by. This self-assurance is no proof of clearness of vision. The day of trial dawns for all, and drink and drugs claim amongst their victims those who have prided themselves on possessing steady heads and a firm purpose. The safeguard is to remember that a patient can never be her own physician. When, therefore, a nurse falls sick, let her consult a medical man, and carry out his prescription loyally, and not attempt to dose herself with remedies as pernicious wrongly used as they are valuable under the right conditions.

### DISTRICT NURSING AT BLACKPOOL.

It is not uncommon, though extremely unpleasant, to find oneself on the floor between two stools. This is the position of the Blackpool Ladies' Sick Poor Nursing Association. The society is carefully organised, and has been the means of doing good work amongst the poor. It is supported by voluntary contributions, which are said to have grievously suffered from the idea which had gained currency that any surplus from the Diamond Jubilee Commemoration Fund was to be given to the association, This surplus, amounting to £180, however, now lies banked in the names of the trustees, who are in no hurry to hand it over to the association. The moral is very clear, namely, that charities should take care to maintain an adequate subscription list, and keep extraordinary sources of income for extraordinary needs.

### SHORT ITEMS.

Dr. Norman Kerr has written to the *Times* urging all friends of the habitual drunkard to rally to the support of the Home Secretary and the Government in their attempt to deal judiciously with what is at least an important part of the great question of habitual drunkenness. He insists, however, at the same time, that provision is equally required for the non-criminal habitual drunkard.

### Antiseptics for Nurses.

By a Medical Woman.

### IX.—ANTISEPTICS DERIVED FROM PHENOL.

There is no doubt that carbolic acid is much less used than it used to be, and is being replaced by other products of coal distillation, which are akin to, but not so irritating nor so toxic in their effects as carbolic acid. This is certainly a step in the right direction, and it is to be hoped that soon now a perfect antiseptic will be found. It is frequently declared that many of the new preparations of coal tar are harmless and even non-poisonous, but this is not the fact, and in spite of all statements to the contrary none of the preparations of coal tar are harmless when taken internally in any but the most minute doses. A case in point occurred not long ago when the death of a woman by asphyxia occurred from drinking a quantity of one of these tar acid preparations, called by the manufacturers "Germol" and described by them as non-poisonous. The truth is that coal tar preparations are only slightly less poisonous than carbolic acid, and consequently all of them should always be labelled and treated as poisonous without any distinction whatever. Dr. A. E. Harris, the medical officer of health for Islington, in his report brings forward some weighty objections to the use of carbolic acid as a disinfectant in the sick room on account of the drying effect it has on the throat and lungs. He further says, "I am now about to trace for you the death record from poisoning by carbolic acid, and I venture to assert that never in the history of any poison has such a silent, persistent, and deadly headway been made without a great public outcry." Dr. Harris then proceeds to prove his assertions by means of statistics, and states that the first recorded death from carbolic acid occurred in 1864, when a woman was accidentally poisoned, and one case is also recorded in each of the succeeding years, and in 1868 the number advanced rapidly to seven for the year. A fact that is very startling is that in 1868 the Poisons Act was passed "to prevent certain poisons being sold unless the purchaser were known to or introduced by some person known to the seller." By some strange oversight, however, carbolic acid was not included amongst these poisons, but was allowed to be sold freely and without restriction, not only by chemists and druggists, but even by grocers and at oilshops. The number of deaths due to carbolic acid rose steadily year by year until in 1875 we reach a record year, and a curious coincidence is pointed out by Dr. Harris, for no one can regard it as cause and effect, namely, that in this same year the Public Health Act was passed. We here give Dr. Harris' table of deaths from carbolic acid by accident and by suicide during the course of seven quinquennial periods as follows:—

| | Deaths by Accident. | Deaths by Suicide. |
|---|---|---|
| 1861-1865 | 2 | 0 |
| 1866-1870 | 18 | 7 |
| 1871 1875 | 86 | 42 |
| 1876 1880 | 100 | 81 |
| 1881-1885 | 111 | 191 |
| 1886-1890 | 127 | 215 |
| 1891-1894 | 129 | 420 |

It is further noticeable that whereas suicidal deaths from all other poisons in the years 1890 to 1894 only increased by 8 per cent., suicidal deaths from carbolic acid alone increased by 95 per cent. Moreover, a larger number of women adopt this method of suicide than do men, in the proportions of 22·36 per cent. men to 35·69 per cent. women. By the above showing the sooner carbolic acid is scheduled as a poison, and a safer antiseptic introduced for general use, the better.

One of the most satisfactory of the more recent coal tar derivatives is called "lysol." It is described as a "tar oil mixed with a fat or fatty oil, and saponified with an alkali, but there is no free alkali. It contains 51 per cent. of volatile oils, consisting of the higher phenols and no carbolic acid, and is thus a mixed disinfectant." It exists, in its undiluted state, as a brown, transparent, oily-looking, clear fluid, with a feebly aromatic odour like creosote. It is only really useful when used in solution, but it is very soluble, and mixes readily with water, and forms a slightly turbid soapy fluid if ordinary hard tap water is used, as it precipitates the lime salts in the water, but is clear if distilled water, alcohol, or glycerine be mixed with it. Under no circumstances, however, are its antiseptic properties interfered with. For surgical purposes a 2 per cent. solution is used. It is reported to be superior as a microbicide to carbolic acid, creolin, cresyl, and other analogous products; but its chief advantage over the antiseptics of established reputation lies in the small amount of irritation or astringent action it produces. It acts to all intents and purposes like a soap, and gives a soapy feel to the hands, which, however, is at once removed by rinsing in sterilised water. It removes all dirt, fatty, or resinous spots from the skin as well as from linen, instruments, &c. It seems well adapted for use in surgical operations, and is sufficiently active and very cheap.

Geosote is a mixture of valerianic acid and creosote, and, hence, is practically a coal-tar preparation. It occurs as a yellow, oily liquid having a sweetish taste, but not the burning character of creosote; it is not very soluble in water, but readily in alcohol, chloroform, and ether. It is not by any means a strong antiseptic, but is useful internally in cases of phthisis, tuberculous joints, &c.

Sozal is another antiseptic prepared from some of the phenols. It is said to be a bactericide, having the advantages in surgery that are possessed by corrosive sublimate without its toxic properties. It occurs in small crystals, which have a strong astringent taste and a slight odour of coal-tar. The crystals are readily soluble in water, glycerine, and spirit.

Piscol and Piscine are other preparations of tar oils; both readily mix with water, and are said to be of the same character as lysol, but cheaper, and not quite such powerful germicides.

Saprol is a tar compound containing both phenol and cresol. It is a dark brown oily fluid, smelling exactly like crude carbolic acid; but it does not mix with water, but divides up into oil drops, some of which sink to the bottom of the vessel while others float on the top of the water. Hence it is not useful for surgical purposes, but acts well as a disinfectant of fæces and other putrescible matter, covering them completely and making a coating through which no evil odours can penetrate, and if left for a sufficiently long time in contact with the matter, saprol will gradually diffuse through it until it has completely pervaded it, and destroys all organisms. It is a very powerful disinfectant, and destroys the more resistant bacilli.

### Royal British Nurses' Association.

A special meeting of the General Council of the Royal British Nurses' Association was held at the rooms of the Medical Society of London, 11, Chandos Street, W., on Friday, the 24th inst, for the purpose of considering the nominations for the new Council. Mr. T. Pickering Pick occupied the chair. The 30 medical men, 30 matrons and superintendents of nurses, and 30 sisters and nurses which the Executive Committee had nominated as elected members of the new Council, and whose names will be submitted to the members at the annual meeting in July for election, were adopted *nem. con.*

### Nursing in Paris Hospitals.

### B.—TRAINING LAY NURSES.

### VIII.—DOCTORS AND NURSES.

THE quotation in Article VI. of this series from the impressive discourse to the graduating midwives at La Maternité by the chief surgeon is an altogether exceptional instance. The midwives are a special class of nurses, and this discourse was at their great fête. At the delivery of diplomas in the ordinary nursing schools the members of the medical and surgical staff of the Paris hospitals, Dr. Bourneville excepted, rarely put in an appearance. Most of the annual speeches are by municipal politicians, hospital directors, or members of the administration of the Assistance Publique.

The conflicting authority of doctors and directors in regard to the nursing staff forms the most dramatic episode in M. Pierre Decourcelle's most popular of recent Parisian plays, "Les Deux Gosses," in English called "Two Little Vagabonds." That such conflicts in Paris are not unknown, and, in fact, have been far from rare, is extremely probable, otherwise the idea in the drama would not have been likely to appeal to either the author or his public. There is, however, great difficulty in getting either class of hospital magnates to acknowledge any such difficulties, or to refer to them. I have minutely interviewed about all the hospital directors and many of the doctors and surgeons, but have never succeeded in extracting much attention to the matter.

Of course, I have here no concern with the relations between hospital directors and the doctors, except as relating to the nursing staff, and the dealings of the doctors with the nurses themselves.

The *mot d'ordre* among the doctors concerning the lay nurses seems to be of recent years typified by the answer given by M. Clemenceau as his defence in a somewhat ludicrous debate raised in Paris socialistic circles recently as to the respective religious compromising practices of three anti-clerical champions—M. Clemenceau himself, and MM. Rochefort and Jaures. As most well-informed people are aware, besides being a maker and unmaker of cabinets, and great republican autocrat, M. Clemenceau is a doctor, and has been a hospital physician. In this latter capacity he has been accused of saying a good word for the Sisters. To this terrible crime in the eyes of militant secularists, M. Clemenceau, in his latest journal, the *Aurore*, replied by explaining that the Sisters and lay nurses contain alike good and bad, selfish and self-sacrificing, competent and incompetent. The astute dialectician argued that the dress made not the least difference; that the Sisters were born into the world nude, that is to say they are women before being Sisters, and have not ceased to be women because they choose to wear a peculiar costume; moreover, that the lay nurses may have all the virtuous qualities even though they do not parade their self-sacrifice by assuming a certain garb.

In varied phrase, and less finely put perhaps, this is the substance of the opinions you will at present extract from most of the hospital doctors concerning the lay nurses and their comparative merits with the Sisters. Some of these opinions as concerning the Sisters I shall have occasion to refer to in the concluding series, when I deal with the subject of the Sisters generally. Meanwhile, I would suggest that the above is only a makeshift opinion after all, and does not touch the root of the matter. It is not a question of dress or of individuals, but as to whether the discipline of the Sisterhood or the individual independence of the lay nurses most induces to the efficiency of hospital nursing services.

This tolerant treatment of the new nurses by the medical staff has not always been the rule. In the first series of articles I referred to the protest, signed by so many hospital physicians and surgeons, against the progress of laicisation. When, in spite of this protest, the laics were introduced, some of the doctors showed not much good sense or good taste in their treatment of the new staff. It is an oft-repeated incident of those days that one well-known champion of the Sisters, chief, surgeon of one of the most important hospitals, in his impatience addressed one of the new nurses as "You filthy laic." The nurse happened to be a woman of spirit, and retorted, "No more filthy than yourself, surgeon!" and she became one of the most trusted and esteemed of the surgeon's assistants.

The incident is illustrative of the early spirit in which the medical and surgical staff greeted the lay nurses except in a small minority; and even to this day the bulk of the doctors and surgeons hold aloof from any active assistance in helping the nurses to qualify for their work. I imagine the doctors and surgeons look with some disdain on what they probably consider as poor amateur attempts at studies in pathology and surgery in the curriculum of the nurses' schools. Certainly many of the doctors throw all the obstacles possible in the way of nurses acquiring varied experience in their profession. I have referred to the necessity found of transferring nurses during the vacation months, so much opposition to such transfers being shown by the doctors while in residence. Certainly without such transfers no nurse could expect to have anything like a complete practical education in the various departments of her trade. There is now no outward show of disregard for the lay nurse in the professional staff of the hospitals, but the nurses are expected to "keep their distance," and not aspire too much. Such education as the nurses receive is gathered from their own chiefs, and by keeping their eyes open.

Of course that large and sometimes mischievous body, nowhere so large and so mischievous as in Paris, the medical students, play a large part in all hospital affairs. So far as the relations of the students with the nurses are concerned, I can only say that it is rare that any difficulties or scandals concerning medical students and nurses are brought to light, and the utmost harmony seems to prevail. This is the more strange, as the Paris medical students are ever to the fore in all sorts of disturbances of public and private tranquillity.

EDMUND R. SPEARMAN.

### Minor Appointments.

THE WESTMINSTER COTTAGE HOSPITAL, SHAFTESBURY, DORSET.—On June 21st Miss L. H. Harris, who received her training at Queen Charlotte's Hospital, was appointed Nurse-Matron of this hospital. She has since had experience in private work, and has passed three years in the employment of the Marylebone Infirmary. She has on two occasions acted as temporary matron of the hospital to which she is now appointed.

### Queen Victoria's Jubilee Institute for Nurses.

Her Majesty has been graciously pleased to approve the appointment of the following as "Queen's" Nurses, to date from July 1st, 1898:—

England.

Superintendents. — Henrietta E. Ellis, Cumberland Nursing Association; Mary White, Northumberland C.N.A.; Fanny Scott, Leeds; Frances Bridgman, Birkenhead; Bertha W. Hall, Huddersfield; Florence A. Grove, Rochdale; and Lydia G. Milne, Ashton-under-Lyne.

Nurses.—Annie C. E. Graham, Emma G. Williams, Emily F. Mantell, Laura Cripps, Amelia E. Hastings, Mary A. Walker, Florence J. Bangham, Mary L. Stephens, Vernie B. Whiting, and Catherine Woodley, London; Amelia M. Sugden, Woolwich; Mary K. Fleetwood, Hampton-on-Thames; Catherine Wise, Turner's Hill; Elizabeth E. Maybrick, Spalding; Mabel Kent, Royston; Rebecca R. Piercy, Bridgwater; Kate H. Wheatley, Morley; Georgiana E. Spencer and Marian F. V. Clarke, Gosport; Ann S. Roe, Warwick; Mary A. Thornber and Mary A. Nedwill, Barrow-in-Furness; Clara E. Homer, Reddish; Bertha Ashburner, Minchinhampton; Edwina Atkins, Stonehouse; Annie Montgomery, Loughton; Alice Dyer, St. Austell; Florence J. Williams, Wribbenhall; Flora Burkinshaw, Hugglescote; Ethel M. Bedell, Rochdale; Frances G. Hawkins, Melbourne; Julie A. Jacobs, Cheltenham; Evelyn Welch, Portsmouth; Florence M. Webb, Leeds; Frances E. Fletcher and Florence M. Vigor, Alnwick; Edith M. Potter and Florence I. Claringbold, Gloucester; Jane Jones, Bolton; Lucy M. Brown and Alice M. Newstead, Kettering; Kate L. Gibson and Florence Cooper, Sunderland; Annie M. Smith and Amice Orme, Birkenhead; Florence M. Ford and Hannah Perry, Birmingham; Jennie A. Cross, Emmie M. Saunders, Beatrice J. Geary, Mary E. Jones, Sarah A. Emby, Annie M. Brown, and Ada Stanley, Manchester; Mary E. Pickering and Sarah E. Neill, Ashton-under-Lyne; Beatrice E. Priestley, Grimsby.

Wales.

Nurses.—Helena Holmes, Cardiff; Jenny Crigan, Treorchy; Lucy James, Kilgerran; Helena Woodworth, Newtown; Catherine Crabb, Welshpool; Janet Hunt, Towyn; Margaret A. Rees, Llanfairfechan; Jessie Williams, Bethesda; Sarah Edmunds, Menai Bridge.

Scotland.

Nurses.—Mary S. Brown, Jessie C. Paul, and Jane Douglas, Edinburgh; Susannah Collins, Agnes Kelly, Bessie Haslam, Lizzie B. Carmichael, Jeannie Mackenzie, and Julia Michael, Glasgow; Jessie A. G. Clark, Aberdeen; Annie G. Spence, Dundee; Annie Telford, Ballantrae; Elsie B. Low, Dumfries; Emily French, Inverness; Jessie L. Neilson, Elgin; Dora M. Walmsley, Kilmarnock; Annie C. Clarke, Stirling; Agnes B. White, Kirkcaldy; Mary W. Coats, Tarbert; Jemima M. Elliot, Tobermory; Alice Dewart, Islay.

Ireland.

Nurses.—Sarah M. Barry and Isabella J. Wallace, Dublin; Alicia Trimble and Kathleen Macgarry, Londonderry; Jennette H. Hargrave, Banbridge; Lucie Woods, Buncrana; Julia Blake, Dundalk; Alicia M. B. Keaghey, Coleraine.

### Where to Go.

Queen's Nurses will be glad to hear that the Princess Christian will distribute badges and certificates to "Queen's" Nurses at Grosvenor House on July 7th, at half-past three p.m.

### Our American Letter.

At the annual meeting of the Nurses' Associated Alumnæ of the United States and Canada, held in New York City, the question of having permanent headquarters in that City was fully discussed, and it was decided that it was to the advantage of the society to have them.

The Alumnæ Association of the Training School for Nurses of the Long Island College Hospital met for its third annual meeting at the rooms of the Y.W.C.A., Brooklyn, New York. The proceedings began by a well-served luncheon, at which twenty-five nurses were present. Miss Ida Craig was appointed president for the ensuing year.

The Associated Alumnæ of the Brooklyn Training School for Nurses have reorganized the office work of registration. Each member has been asked to canvass her own school with a view to finding out the opinion of the majority as regards the amount of the registry fee. It appears probable that one of ten dollars will meet with the greatest approval.

Mr. William L. Bull, of Whitford, has given £2,000 (10,000 dollars) to build and establish a training school for nurses in connection with the Chester County Hospital, West Chester, Pa. The school will probably be commenced shortly.

Accommodation for 1,000 sick has been secured by the American Government at Key West. The convent of the Immaculate Conception and two large factories are arranged as hospitals. The nursing is undertaken by the Sisters, who give their services in return for maintenance and clothing. A small portion of the convent is reserved for their own use.

### A Woman's Hospital in Morocco.

The Countess of Meath has a most interesting article in the June *Nineteenth Century* on the first woman's hospital in Morocco. The hospital is in Tangier, and is necessarily under the charge of a medical woman, who, with her small nursing staff, has at last overcome the many difficulties and native prejudices which at first seemed insurmountable.

The furnishing of a Morocco hospital is not an expensive undertaking, since Lady Meath tells us the waiting-room of the Tangier Hospital is absolutely guiltless of furniture of any kind. It has a wall and floors, on which the women squat, On one occasion a patient was admitted to a room whose wall was adorned by a picture of the Prodigal Son. On seeing it she instantly covered her face with the folds of her "haik," a blanket in which she was enveloped, and refused to sleep in a room wherein was the picture of a man!

The early patients to the hospital needed much courage, since they were assured by friends and neighbours that they would either be poisoned, cut to pieces, or turned into "Nazarenes" by being compelled to eat pork. Patience and perseverance have overcome all these prejudices, and the patients flock to the Tabeeba, that is, the lady doctor.

The dieting of a Morocco hospital should prove neither troublesome to the staff nor burdensome to the exchequer. Breakfast consists of bread with very weak and much sweetened coffee, while bread and raisins form the midday meal. The principal repast, served at four p.m., consists of meat, vegetables, and rancid butter boiled together. During the great Mohammedan festival of Ramadan not a morsel of food nor a drink of water may be given to the sick, and all medicines must be taken during the night while the Ramadan lasts. Their symptoms, as described by the Moors, are distinctly involved. One woman described herself thus: "My head is imprisoned; it is all contracted. Sometimes I have no eyes. I am dead and cannot raise myself off my bed. Something got into my head, went down to my chest, from there to my stomach, and into my legs." The article is well worth reading.

### Training in the Provinces.

(*Continued from page* 117.)

### BOOTLE BOROUGH HOSPITAL (103 Beds).

### Terms of Training.

PROBATIONERS are received at the Bootle Borough Hospital for a period of two years' training. Candidates should be between 22 and 35 years of age; one month's trial is required before acceptance. No premium is expected from probationers, but no salary is given to them for the first year, and they are required to provide their own uniform; at the end of the first year the sum of one guinea being paid to them "as the probable cost of the same." For the second year probationers are paid £12, and a certificate is given at the satisfactory completion of the engagement and passing of a final examination. Lectures are given to the nurses during their two years' training by the senior house surgeon. Two years' training in the medical and surgical wards of the hospital, on day and night duty, are the necessary qualifications for appointment to the charge of wards. Staff nurses are paid £25 and sisters £30 per annum.

During the two years every probationer is required to do six months' night duty, that is, three months in each year. There is a permanent night sister.

### Hours of Work and Times off Duty.

Probationers begin duty in the morning at 7 and leave their wards in the evening at 8.30. Two or three hours are allowed daily for recreation, and half an hour each for luncheon, dinner, and tea. One whole day off is given each month, on which day the nurses have no duty in the wards at all. Probationers get a fortnight's leave, staff nurses three weeks, during the year.

### Meals.

All meals are served in the nurses' dining-room, no "stores" being given out to the staff. The hours for meals are as follows: Breakfast, 6.30 a.m; luncheon, 9.30 a.m.; dinner, 12.45 to 1.15 and 1.15 to 1.45 p.m.; tea 4 to 5 p.m.; supper, 9 p.m.

### BLACKBURN INFIRMARY (102 Beds).

### Terms of Training.

Candidates for training at the Blackburn and East Lancashire Infirmary must be between 21 and 30 years of age. A month's trial is required before final acceptance, and if this is satisfactorily gone through, a three years' engagement is entered upon. A premium of £10 10s. is asked of probationers, salary commencing at once, with £10 for the first year, £18 for the second, and £20 for the third, rising subsequently to £30. Sisters are paid £28 per annum, increasing to £34 by £2 annually. Laundry and indoor uniform are provided.

Promotion to the responsible posts of charge-nurse or sister depends upon the competency shown for management, a three years' certificate being an essential qualification.

Night duty is taken alternately with day duty, three months being the length of night work taken at a time.

### Hours of Work and Times off Duty.

Probationers go on duty in the morning at 7, having a light early meal previously, and their regular breakfast an hour and a half later. The day staff leave the wards in the evening at 8.30 o'clock, all nurses having one hour off duty during the afternoon. Half an hour is allowed for each meal. Charge nurses do not go on duty in the morning till 8.30, having their breakfast first. They leave their wards in the evening at the same time as the probationers, and their hours otherwise are similar. The day nurses and probationers have one half-day during the week, in addition to a half-day on alternate Sundays, from 2 to 9.30 in summer, and 2 to 9 p.m. in winter. Besides this they may have leave for church on alternate Sunday mornings and evenings. Night nurses are on duty from 8.30 a.m. to 8.30 p.m., being off duty each day between their dinner at 9.15 p.m., and going to bed at 1 p.m.

Three weeks' holiday is allowed at the end of each complete year of service.

Lectures are given to the nurses by the senior house-surgeon and the matron (Miss H. C. Poole) upon anatomy and physiology, medical and surgical nursing, feeding and care of the sick.

### Meals.

Meals are all served in the nurses' dining-room. As mentioned before, the day probationers begin the day with a light, early breakfast of tea and bread and butter at 6.30, breakfast being served at 8 30, dinner at 12, tea between 4 and 5, and supper at 8.30. Charge-nurses breakfast at 8 a.m., have their dinner at 12.30, tea at 4.30, and supper at the same time as the probationers.

Night nurses breakfast at 8 p.m., and dine at 9.15 a.m.

The only "stores" given out to the nurses are to those on night duty.

### BRADFORD ROYAL INFIRMARY (220 Beds).

### Terms of Training.

Applicants for training at the Bradford Royal Infirmary should be between 21 and 32 years of age. If accepted after a month's trial a three years' engagement is entered into, certificates being given on its satisfactory completion. No salary is offered during the first year, £16 are given during the second, and £20 during the third year. Salaries given subsequently to nurses on the permanent staff vary between £25 and £30. Laundry is provided and indoor uniform. No "special" or paying probationers are received at this training school.

Theoretical instruction is given to the nurses by members of the visiting staff, and lectures on nursing subjects by the night superintendent, examinations being held and medals presented to the most successful.

The full term of three years' training is an essential qualification for promotion to the post of charge nurse or sister of wards, the latter appointment being given only to those who show special aptitude for the duties of this responsible position. Probationers in the second year take night duty for about nine consecutive months.

### Hours of Work and Times Off Duty.

The working day for probationers is from 7 a.m. to 8 p.m., with the usual time allowed for meals, and two hours "off" every day for all the staff. Three hours' leave is given on Sundays, half a day once a fortnight to sisters, and half a day once a month to probationers. Fourteen days' annual leave is allowed at present, but the whole question of the hours and holidays of the nursing staff is now under the consideration of the committee, and it is hoped that arrangements will be made for a whole day off once a month, and that the yearly holiday will be increased to not less than three weeks.

### Meals.

All meals, including luncheon and tea, are served in the dining hall, no "allowances" being given out to the nurses. The hours of meals for the day staff are as follows: Breakfast, 6.30 to 7 a.m.; lunch, 9 to 9.30 and 9.30 to 10 a.m.; dinner between 12.30 and 2 p.m.; tea, 4 to 5; supper, 8 to 8.30 and 8.45 to 9.15 p.m.

### Everybody's Opinion.

[Correspondence on all subjects is invited, but we cannot in any way be responsible for the opinions expressed by our correspondents. No communication can be entertained if the name and address of the correspondent is not given, as a guarantee of good faith but not necessarily for publication, or unless one side of the paper only is written on.]

MATRONS' COUNCIL MEETING.

"A Constant Reader" writes: It is mentioned in a paper read at the above meeting that probationers cannot get proper training in a hospital of 40 beds. Speaking from past experience, I think it only fair to those now in work in such to say this is somewhat hard and false. I have had probationers in a country hospital which has turned out good nurses who could take any case; they have taken good work in London afterwards, and been complimented by a London matron on their knowledge. In a country hospital the poor probationers who learn nothing have the chance of seeing all cases —medical and surgical operations—knowing how the preparation of the theatre and instruments should be arranged; and they have to do dressings during their training, instead of seeing the students always doing them. In my training in London I was taught "Practice makes perfect." Is it only passing examinations and serving in a big London hospital three or four years that makes a good nurse now? I fear not. I know some who have done this, and who yet have not seen many cases nor nursed patients that many country probationers have helped to nurse. I am sorry for the small hospital matrons and nurses if they can teach nothing to the poor probationers, and why should the time spent by them be counted for nothing and be looked down upon by the London matrons? What are the country poor to do when ill, for soon no nurses will go to such places? I wish others would give their experience on this.

### Appointments.

MATRONS.

City and County of Kingston-upon-Hull Sanatorium. —Miss Agnes F. Abbott was appointed Lady Superintendent of this institution on June 23rd, 1898. She was trained at the Children's Hospital, Great Ormond Street, London, and St. Bartholomew's, London, and held the post of sister at the Clinical Hospital, Manchester, from 1884 to 1891; from April, 1891, to January, 1893, held the post of matron at the Southern Hospital, Manchester; and since January, 1893, has held appointment of lady superintendent at Wirral Children's Hospital, Birkenhead.

Cuddington Isolation Hospital, Surrey.—On June 27th, 1898, Miss Alice Mary Raynes was appointed Matron of this hospital. She was trained for three years, and was afterwards staff nurse at St. George's Hospital, Hyde Park Corner, W.; at which hospital, after having been charge nurse of the South-Western Fever Hospital, Fulham, for a few months, she held the position of assistant matron.

Sussex, Brighton, and Hove Ear and Throat Hospital, Brighton.—Miss Evelyn Hilton has been appointed Matron of this hospital. Miss Hilton trained at Newark Hospital and at Charing Cross Hospital, and since then has held the appointment of assistant matron at the Birmingham and Midlands Eye Hospital, Birmingham.

### Presentations.

Miss Annie Hamilton, on leaving the Royal United Hospital, Bath, was presented with a gold watch and a testimonial from the committee and staff; a case of silver apostle teaspoons and sugar tongs from the matron, resident medical officer, and resident surgical officer; and a gold ring and afternoon tea service from the nurses.

Huddersfield and Halifax Nurses' Homes.—On the occasion of Miss Helen Murray's marriage at Halifax on June 18th, the staffs of the two homes each presented her with a handsome piece of plate.

### The Book World for Women and Nurses.

[We invite Correspondence, Criticism, Enquiries, and Notes on Books likely to interest Women and Nurses. Address, Editor, The Hospital (Nurses' Book World), 28 & 29, Southampton Street, Strand, London, W.C.]

A Healthy Home and How to Attain It. By Dr. Andrew Wilson. (London: C. A. Pearson, Limited. 1898.) Pp. 86. Price 1s.

This little volume is the fourth of a series, designed for the use of young housekeepers, and now being issued from the office of *Home Notes*. Dr. Wilson has written it—as he always writes—pleasantly and lucidly, yet without the sacrifice of accuracy so common to "popular" manuals. The class to whom it is addressed is perhaps the class which above all others is resentful of advice and tenacious of domestic conventionalities. But this little book is written in precisely the vein which will most attract and influence them. There is nothing in the subject matter which calls for adverse criticism. Dr. Wilson's suggestions are made with taste and wisdom, and with due regard to financial limitations. Perhaps distempering, as a method of wall-decoration, should have been mentioned. It is, in our opinion, preferable to either papering or oil-painting, since it is cheap, clean, easily renewed, and of artistic possibilities which are only limited by the taste of the householder. We note the approaching issue of a companion volume—on Food and Drink—by the same author.

About Children: Six Lectures given to the Nurses in the Training School of the Cleveland General Hospital in February, 1896. By Samuel W. Kelley, M.D., Professor of Diseases of Children in the Cleveland College of Physicians and Surgeons, &c., Cleveland. (The *Medical Gazette* Publishing Company. 1897.)

Dr. Kelley's lectures on children and their diseases form a most interesting as well as valuable contribution to a perennially interesting subject. He warns his listeners against regarding child patients as the same as grown-ups, only smaller, and notes the various points of difference. Very few nurses who have not undergone some training in a children's hospital fully appreciate these differences, and still fewer mothers are aware of them. The rapid variations of children's temperatures form a frequent cause of alarm to an anxious mother, who has invested in a clinical thermometer, and gets frightened at a rise in temperature, without inquiring into its possible cause. In fact, all the chapters on the development of infants should be read by mothers; it might quell some fond parent's pride in the precocity of her offspring, but it will help her largely to appreciate the meaning of the various sounds and movements made by her baby, and guide her as to when she should regard these with satisfaction and when with anxiety. In treating of the various ailments and accidents of childhood, Dr. Kelley shows the same clearness, scientific knowledge, and practical sagacity; while the pungent vividness of his style and the brightness of his illustrations make his book one that is eminently suitable for popular reading, while they in no way detract from its value as a book for serious study.

A very charming "Evening Hymn for Hospital Nurses," by the Rev. Harvey Gem, is before us for review, a hymn of ten verses set to music by the well-known Dr. Varley Roberts, organist of Magdalen College Chapel at Oxford. This hymn, which is dedicated by permission to H.R.H. Princess Christian of Schleswig-Holstein, has been lately published and printed in a convenient-sized leaflet by Henry Froude, Oxford University Press Warehouse, Amen Corner, E.C., and it can be obtained of all booksellers at 2s. per dozen. We feel assured that the words which Dr. Roberts has so admirably set to music will commend themselves to the notice of all serious-minded nurses for their evening use.

### For Reading to the Sick.

### OUR SURE REWARD.

Come, labour on,
The toil is pleasant, the reward is sure,
Blessed are those who to the end endure;
How full their joy, how deep their rest shall be
O Lord with Thee.
—*J. Borthwick.*

Thy cross is quite enough for thee,
Though little it appears,
For there is hid in it the weight
Of the eternal years.
He practises all virtue well
Who his own cross reveres,
And lives in the familiar thought
Of those eternal years. —*F. W. Faber.*

Go thou into thy closet: shut thy door,
And pray to Him in secret: He will hear.
But think not thou by one wild bound to clear
The numberless ascensions, more and more,
Of starry stairs, that must be climbed, before
Thou comest to the Father's likeness near;
And bendest down to kiss the feet so dear,
That step by step their mounting flights passed o'er.
—*George Macdonald.*

"He saw with Faith's far-reaching eye the fount
Of Life, his Father's house, his Saviour, God,
And borrowed thence to help the present want . . .
And so his eye upon the Land of Life
He kept. Virtue grew daily stronger, sin
Decayed; his enemies, repulsed, retired;
Till at the stature of a perfect man
In Christ arrived, and with the Spirit filled,
He gained the harbour of eternal rest."
—*Pollok.*

Teach me to live, with kindly words for all,
Wearing no cold, repulsive brow of gloom—
Waiting with cheerful patience till Thy call
Summons my spirit to her heavenly Home.

Thou hidden source of calm repose;
Thou all-sufficient love divine;
My Help and Refuge from my foes,
Secure I am if Thou art mine;
And lo! from sin and grief and shame
I hide me, Jesus, in Thy name!

Thy mighty Name salvation is,
And keeps my happy soul above;
Comfort it brings and power and peace,
And joy and everlasting love;
To me with Thy dear Name are given
Pardon and holiness and heaven.
—*Charles Wesley.*

**Reading.**

Be ye of good cheer, every one that is afflicted, for the Lord is preparing for you the City of God. Whatever be your sorrow, it is the token of His love; for the Man of Sorrows is our King, and the path of sorrow is the path of His Kingdom; there is none other that leadeth into life. Your reward is sure if you are but true to yourself. Do we believe these things? Are they realities, or are they words? They are God's Word, which is reality.—*Dr. Manning.*

Meditate long, meditate humbly on what it is to have a Creator, and comfort will come at last. If broad daylight should never be yours on this side of the grave He will hold your feet in the twilight that they shall not stumble, and at last with all the more love and all the more speed as well, He will fold you to His bosom, Who is Himself the Light Eternal.—*F. W. Faber.*

### Notes and Queries.

*Mental Nursing.*

(125) Can you give me any hints on mental nursing? I have had some experience, but cannot get away to attend lectures often. Is there a course of study that I could go through by attending lectures, say, once a month in London?—*Mental.*

You should have sent name and address. The Registrar, the Medico-Psychological Association, 11, Chandos Street, W., could best help you.

*Marking Ink.*

(126) Kindly tell me the best ink for marking linen. Where can it be obtained?—*M. Robison.*

"Nigrine" is very good. Whiteley's, Westbourne Grove, used to keep it. Bond's marking ink is an old favourite and still holds its own. You can get it nearly everywhere.

*Self-Consciousness.*

(127) Please advise how to overcome self-consciousness. Perpetua shyness is a great pain, both to the sufferer and others, especially in hospital work.—*Nurse Newly entered.*

The only cure for self-consciousness is humility and self-forgetfulness. Humility to remember that one is only a new "pro," and of not much account in the busy hospital life, and to forget oneself in taking pains to understand one's own work and to sympathise with every one else's troubles and difficulties.

*Superfluous Hair.*

(128) Will you kindly tell me of a reliable establishment in London where superfluous hairs can be permanently removed?—*Anxious.*

We cannot recommend any establishment where superfluous hairs are removed, because electrolysis being a surgical operation appertains to the work of a specialist on skin diseases. You should, therefore, consult such a one or your own medical man.

*Weir Mitchell Treatment.*

(129) Can you tell me of a home, hospital, or other institution where a working man, suffering from long-standing nervous illness, could be received? He has been treated without avail under all the local medical charities, and is incapacitated from earning his living. His medical man will give any information that may be required.

The National Hospital for the Paralysed and Epileptic, Queen's Square, Bloomsbury, might help the case. It would also be worth while trying, if this failed, to obtain his admission into one of the wealthier London hospitals, where every possible means of cure would be provided.

*Testing and Fevers.*

(130) Can you tell me of a good book on testing and fever nursing? Are appointments published free?—*A. C.*

Dr. Harding's "Fevers and Infectious Diseases; their Nursing and Practical Management." Price 1s. (Scientific Press, London). We are always pleased to receive early notices of appointments, which we publish free. The training and career of the person appointed should be stated.

*Training as Parish Nurse.*

(131) Where could I get a thorough knowledge of general and monthly training in the shortest time in order to take up parish work? I could not afford to give time.—*An Inquirer.*

It is impossible to get a thorough knowledge of general nursing in a short period, and if you cannot afford to give time you had much better take up the three years' training at a good training school, where you will not only receive very fair wages, but at the end of your training a certificate which will give you a chance of obtaining really good positions. Your letter is dated from South Wales. The General Hospital, Swansea, gives a salary of £10 to probationers for the first year, £12 for the second, and £14 the third.

*Medicine and Nurses.*

(132) Kindly recommend a book upon medical diseases and their treatment for private study. 2. Could you give me any information as regards the treatment of corpulency apart from dieting and exercise?—*Nurse B.*

We should advise a nurse to study nursing rather than medicine. There is a good book upon medical nursing by the late Dr. James Anderson, edited by E. L. Lamport (H. K. Lewis), and Messrs. J. and A. Churchill publish "Lectures on Medicine and Nurses," by Dr. Herbert Cuff.

*Board and Lodging.*

(133) Can you tell me of a nursing or Y.W.C.A. home close to St. Martin's Town Hall where a young girl who is to sit for her examination could have a bed-room and board for a week?—*E. W.*

Write to the Secretary, the Victoria (Commemoration) Club, Southampton Street, Strand. She will probably be able to give you help.

*Male Nurse.*

(134) I have good experience as a male nurse and am a total abstainer. How can I get into a London association?—*Subscriber.*

Write to the Secretary, the Male Nurses (Temperance) Co-operation, 10, Thayer Street, Manchester Square, W.

*Naval and Military Nurses for America.*

(135) Seeing in a paragraph in the "Mirror" that nurses are required for the American army and navy, I should like to know more about it, and where I could obtain information.—*L. M. W.*

The American Embassy in London might possibly give you information; you might also write to Dr. Anita McGee, at Washington. In case you think of offering your services we draw your attention to the facts that any number of American nurses are eagerly awaiting a chance of admission into these services and comparatively few are wanted. Judge then how little hope there is of an English nurse obtaining the coveted appointment.

